# Coronary Microvascular Dysfunction: Pathophysiology, Clinical Phenotypes, and Mechanism-Guided Diagnostic Strategies

**DOI:** 10.3390/jcdd13090426

**Published:** 2026-09-01

**Authors:** Divyansh Sharma, Daniel Bishev, Shudipan Chakraborty, Michael V. DiCaro, Ryan Shao, KiBuom Lee, KaChon Lei, Omar Shah, Sanjay Malhotra, Chowdhury Ahsan

**Affiliations:** 1 Department of Cardiovascular Medicine, Kirk Kerkorian School of Medicine, University of Nevada, Las Vegas, NV 89106, USA; daniel.bishev@unlv.edu (D.B.); shudipan.chakraborty@unlv.edu (S.C.); michael.dicaro@unlv.edu (M.V.D.); ryan.shao@unlv.edu (R.S.); sanj6268@yahoo.com (S.M.); 2 Department of Cardiology, University of Arkansas for Medical Sciences, Little Rock, AR 72205, USA; kibuom.lee@unlv.edu; 3 Department of Cardiology, Scripps Clinic, La Jolla, CA 92037, USA; kachon.lei@unlv.edu; 4 New York University, New York, NY 10003, USA; shahomar991@gmail.com

**Keywords:** coronary microvascular dysfunction, ischemia with no obstructive coronary arteries, invasive coronary physiology, coronary flow reserve, index of microcirculatory resistance, vasomotor dysfunction, acetylcholine testing, microvascular angina, stable coronary artery disease, acute coronary syndromes

## Abstract

Coronary microvascular dysfunction (CMD) is a prevalent and clinically important cause of myocardial ischemia across the spectrum of coronary artery disease, including patients with angina and nonobstructive coronary arteries, persistent symptoms after revascularization, and acute coronary syndromes. Despite its prognostic significance, CMD remains underrecognized due to the limitations of angiography-centered evaluation and the heterogeneity of underlying biological mechanisms. This review presents a physiology-based framework for the assessment of CMD, emphasizing the role of invasive coronary function testing in defining mechanistic endotypes and informing clinical interpretation. We summarize the biological foundations of CMD, including endothelial dysfunction, vascular smooth muscle hyperreactivity, structural remodeling of the microcirculation, and extravascular myocardial influences, and describe how these processes manifest as distinct but overlapping clinical phenotypes. Contemporary invasive physiologic tools—coronary flow reserve, index of microcirculatory resistance, and vasomotor testing with acetylcholine—are reviewed with a focus on their complementary roles in localizing disease within the coronary circulation. The clinical application of microvascular testing is discussed in both stable coronary syndromes and acute coronary syndromes, highlighting differences in diagnostic intent, prognostic implications, and therapeutic relevance. We distinguish chronic CMD in stable syndromes from post–myocardial infarction microvascular injury, in which invasive indices have primarily prognostic implications. By integrating coronary biology, invasive physiology, and clinical context, this review supports a shift from purely anatomic assessment toward a mechanism-based, physiology-driven approach to ischemic heart disease.

## 1. Introduction

Coronary angiography has long served as the foundation for the diagnosis and management of ischemic heart disease. By delineating the presence and severity of epicardial coronary artery stenoses, angiography has guided decisions regarding revascularization and informed prognosis for decades. However, angiography provides a luminographic assessment of the coronary tree and offers limited insight into the functional behavior of the coronary circulation. As a result, a substantial proportion of patients presenting with angina, objective evidence of ischemia, or adverse cardiovascular outcomes are found to have nonobstructive or only mildly obstructive coronary artery disease on invasive coronary angiography [1,2,3].

This discordance between symptoms, ischemia, and angiographic findings is increasingly recognized as clinically important. Observational cohorts and randomized studies have demonstrated that patients with angina and nonobstructive coronary arteries frequently experience persistent symptoms, impaired quality of life, recurrent hospitalizations, and excess long-term cardiovascular risk compared with asymptomatic individuals [4,5,6]. These observations challenge the historical paradigm that equates ischemia exclusively with flow-limiting epicardial disease and underscore the limitations of angiography-centered diagnostic strategies.

The Women’s Ischemia Syndrome Evaluation (WISE) program was instrumental in reframing this problem. WISE investigators demonstrated that abnormalities in coronary vascular function—particularly impaired coronary flow reserve and endothelial dysfunction—were associated with adverse cardiovascular outcomes despite the absence of obstructive epicardial disease [4,5,6]. These findings established that ischemia in the absence of obstructive coronary artery disease is neither benign nor incidental, and that coronary vascular dysfunction itself represents a clinically meaningful disease substrate.

Coronary microvascular dysfunction (CMD) has since emerged as a central mechanism underlying ischemia across a wide spectrum of clinical presentations. CMD refers to abnormalities of the coronary microcirculation, including pre-arterioles, arterioles, and capillaries, that impair appropriate regulation of myocardial blood flow in response to metabolic demand. These abnormalities may involve dysfunctional vasomotor signaling, increased microvascular resistance, maladaptive vasoconstriction, or structural remodeling of the microvasculature [7,8,9]. CMD may occur in isolation or coexist with epicardial coronary artery disease, with important implications for symptoms, prognosis, and response to therapy.

CMD is now recognized in several clinically important settings. In patients with angina and nonobstructive coronary arteries (ANOCA/INOCA), CMD and coronary vasomotor disorders account for a substantial proportion of ischemic symptoms. In patients with obstructive coronary artery disease, CMD may coexist with epicardial lesions and contribute to ischemia that is disproportionate to angiographic severity. Following percutaneous coronary intervention, residual or recurrent angina is frequently attributable to persistent microvascular dysfunction rather than procedural failure [10,11,12]. In acute coronary syndromes, microvascular injury may persist despite restoration of epicardial patency and is a major determinant of infarct size, ventricular remodeling, and long-term outcomes [13,14,15].

Despite its growing recognition, CMD has historically been difficult to diagnose. Traditional noninvasive stress testing identifies ischemia but does not define its mechanism, while coronary angiography excludes obstructive epicardial disease without providing direct assessment of microvascular function. Consequently, many patients have been left without a clear mechanistic explanation for symptoms or ischemia, leading to diagnostic uncertainty and empiric management strategies.

Advances in invasive coronary physiology have addressed this gap. Techniques such as coronary flow reserve (CFR), resistance-based indices including the index of microcirculatory resistance (IMR), and acetylcholine provocation testing now permit direct, quantitative assessment of coronary microvascular function and vasomotor behavior in the cardiac catheterization laboratory [10,11,12,13,16]. These tools provide insight into both flow capacity and vasomotor regulation that is not accessible with angiography alone.

Reflecting this evolution, contemporary clinical practice guidelines and expert consensus statements from the European Society of Cardiology (ESC), American College of Cardiology/American Heart Association (ACC/AHA), and Society for Cardiovascular Angiography and Interventions (SCAI) now recognize CMD and coronary vasomotor disorders as clinically relevant diagnoses [14,17,18]. These documents support invasive coronary functional testing in selected patients, particularly those with persistent angina and nonobstructive coronary arteries or unexplained ischemia despite apparently successful revascularization.

However, CMD remains a heterogeneous condition encompassing multiple biological mechanisms and clinical phenotypes. Physiologic indices differ in what they measure and how they should be interpreted, and no single test captures the full complexity of microvascular disease. Without a clear framework linking microvascular biology to physiologic measurements and clinical presentation, invasive testing risks becoming descriptive rather than actionable.

Accordingly, a structured, mechanism-based approach to CMD is needed—one that distinguishes underlying pathophysiologic processes, clarifies the role of individual physiologic indices, and integrates invasive testing into clinical decision-making. The purpose of this review is to provide such a framework. We first examine the biological mechanisms and endotypes of CMD, then discuss how invasive physiologic tools interrogate these processes, and finally explore the clinical implications of microvascular testing in stable coronary artery disease and acute coronary syndromes. By emphasizing physiology as a means of defining mechanism rather than merely confirming ischemia, this review aims to place invasive microvascular testing within a coherent, clinically meaningful context.

## 2. Coronary Microvascular Dysfunction: Biological Mechanisms and Clinical Endotypes

Coronary microvascular dysfunction represents a spectrum of abnormalities affecting the coronary microcirculation that impair appropriate myocardial perfusion. Unlike epicardial coronary artery disease, which is defined anatomically by focal or diffuse luminal narrowing, CMD is fundamentally a disorder of vascular function and structure at the level of the microvasculature. As such, CMD cannot be adequately described by angiography alone and requires a mechanistic framework that integrates vascular biology, myocardial interaction, and clinical expression.

A central challenge in CMD is heterogeneity. Patients with similar symptoms or ischemic burden may have distinct underlying biological mechanisms, while similar mechanisms may manifest differently depending on systemic conditions and myocardial context. For this reason, it is useful to distinguish the mechanistic foundations of CMD from clinical endotypes, which describe how these mechanisms present physiologically and clinically. This distinction provides the conceptual basis for invasive functional testing and underpins contemporary guidelines and consensus approaches to the evaluation of coronary vascular dysfunction.

### 2.1. Mechanistic Foundations of Coronary Microvascular Dysfunction

#### 2.1.1. Endothelial Dysfunction and Impaired Vasodilatory Signaling

The coronary endothelium plays a central role in regulating microvascular tone through the release of nitric oxide and other vasoactive mediators in response to shear stress and metabolic demand. In CMD, endothelial dysfunction is characterized by reduced nitric oxide bioavailability due to oxidative stress, inflammation, and uncoupling of endothelial nitric oxide synthase [18,19]. Reactive oxygen species degrade nitric oxide and impair downstream cyclic guanosine monophosphate signaling, limiting smooth muscle relaxation.

In parallel, increased expression of vasoconstrictor pathways—particularly endothelin-1—promotes heightened basal tone, exaggerated vasoconstrictive responses, and progressive vascular remodeling [19,20]. These abnormalities provide a mechanistic explanation for ischemia occurring at rest or during emotional stress and for paradoxical vasoconstriction observed during provocative testing.

#### 2.1.2. Vascular Smooth Muscle Dysfunction

Beyond endothelial signaling, CMD may arise from intrinsic abnormalities of vascular smooth muscle cells. Altered calcium handling, impaired cyclic nucleotide signaling, and increased basal contractility limit the ability of resistance vessels to dilate appropriately during conditions of increased demand [10,11].

This mechanism is largely endothelium-independent and contributes to impaired hyperemic flow even when endothelial responses appear preserved. Smooth muscle dysfunction is often associated with chronic cardiometabolic disease and may coexist with endothelial abnormalities, contributing to fixed limitations in vasodilatory capacity.

#### 2.1.3. Structural Remodeling of the Microcirculation

Chronic exposure to metabolic, inflammatory, and hemodynamic stress leads to structural remodeling of the coronary microvasculature. This includes arteriolar hypertrophy, luminal narrowing, capillary rarefaction, and perivascular fibrosis, all of which increase minimal microvascular resistance and reduce maximal achievable coronary flow [20,21,22].

Structural remodeling is particularly prevalent in patients with diabetes, hypertension, obesity, chronic kidney disease, and aging. Importantly, these changes are often progressive and less reversible than functional abnormalities, contributing to persistent ischemia and adverse prognosis despite optimal control of epicardial disease [21,22,23].

#### 2.1.4. Myocardial–Vascular Interaction

The coronary microcirculation functions within the mechanical environment of the myocardium. Elevated left ventricular end-diastolic pressure, myocardial hypertrophy, and increased wall stress compress intramyocardial vessels, particularly during diastole, increasing effective microvascular resistance [22,24].

This interaction is especially relevant in heart failure with preserved ejection fraction, where CMD is increasingly recognized as a central pathophysiologic mechanism linking systemic inflammation, endothelial dysfunction, and myocardial stiffness [21,22,23]. In this context, CMD is both a contributor to and a consequence of myocardial dysfunction.

### 2.2. Endothelium-Dependent and Endothelium-Independent Microvascular Dysfunction

Building on these mechanistic foundations, CMD encompasses heterogeneous biological mechanisms that differ not only in pathophysiology but also in physiologic behavior and pharmacologic responsiveness. A clinically relevant distinction can be made between endothelium-dependent and endothelium-independent microvascular dysfunction, as these mechanisms demonstrate differential responses to invasive testing and therapeutic interventions [7,8,9].

Endothelium-dependent microvascular dysfunction is characterized by impaired nitric oxide bioavailability, heightened oxidative stress, and increased vasoconstrictor signaling, particularly via endothelin-1 pathways [7,8]. In this phenotype, intracoronary acetylcholine typically provokes an abnormal vasoconstrictive or blunted vasodilatory response, reflecting endothelial dysfunction, while hyperemic flow in response to adenosine may be relatively preserved [17,25]. From a therapeutic perspective, interventions that improve endothelial function—including angiotensin-converting enzyme inhibitors, angiotensin receptor blockers, statins, and lifestyle modification—are biologically plausible and supported by improvements in endothelial signaling rather than reversal of fixed microvascular disease [8,14].

In contrast, endothelium-independent microvascular dysfunction reflects intrinsic abnormalities of vascular smooth muscle relaxation or structural remodeling of the microcirculation, including arteriolar wall thickening, perivascular fibrosis, and capillary rarefaction [7,9]. Physiologically, this phenotype is characterized by impaired hyperemic response to adenosine, elevated microvascular resistance, and reduced coronary flow reserve despite preserved or minimally abnormal acetylcholine responses [10,11,26]. Pharmacologic vasodilators often demonstrate limited efficacy in this setting, and management is frequently directed toward symptom modulation and treatment of contributing myocardial or systemic factors rather than reversal of microvascular pathology [8,20].

Importantly, overlapping features of endothelium-dependent and endothelium-independent dysfunction are common, reinforcing the need for integrated physiologic assessment rather than reliance on a single diagnostic metric [16,27].

### 2.3. Clinical Endotypes of Coronary Microvascular Dysfunction

While mechanistic processes often overlap, CMD is most clinically useful when conceptualized through dominant endotypes that reflect characteristic physiologic behavior and clinical presentation. Endotype classification allows clinicians to move beyond descriptive diagnoses toward mechanism-informed interpretation of invasive testing [14,15,16].

#### 2.3.1. Endothelial-Dependent CMD

Endothelial-dependent CMD is characterized by impaired nitric oxide–mediated vasodilation and abnormal vasomotor responses. Clinically, this endotype often presents with episodic angina, frequently occurring at rest or during emotional stress rather than exertion.

This phenotype is best identified by provocative testing with acetylcholine, which may demonstrate attenuated dilation, paradoxical constriction, or ischemic symptoms with electrocardiographic changes in the absence of significant epicardial narrowing [25,27,28]. Resting angiography and pressure-derived indices are frequently normal, underscoring the importance of vasomotor assessment.

#### 2.3.2. Endothelium-Independent (Structural) CMD

Endothelium-independent CMD reflects fixed microvascular disease characterized by elevated resistance and impaired hyperemic flow. This endotype is closely associated with cardiometabolic comorbidities and structural remodeling of the microcirculation.

Patients typically experience exertional angina with reproducible ischemia. Prognostically, structural CMD is associated with adverse cardiovascular outcomes independent of epicardial disease severity [10,11,12,29]. This endotype is less responsive to therapies targeting vasomotor instability alone, highlighting the importance of mechanistic distinction.

#### 2.3.3. Vasospastic Endotypes

Vasospastic disorders represent a distinct group of CMD endotypes characterized by exaggerated vasoconstrictive responses. Epicardial vasospasm involves transient focal or diffuse constriction of conduit vessels, whereas microvascular spasm produces ischemic symptoms and electrocardiographic changes without angiographically visible epicardial narrowing [25,27,28].

These disorders reflect severe endothelial dysfunction, heightened smooth muscle reactivity, and autonomic imbalance. Recognition is critical, as vasospastic endotypes respond differently to pharmacologic therapy and may be exacerbated by treatments commonly used in structural CMD [14,15,16].

#### 2.3.4. Mixed and Overlapping Endotypes

In practice, many patients exhibit overlapping CMD endotypes. Endothelial dysfunction may coexist with structural remodeling, or vasospastic tendencies may be superimposed on diffuse microvascular disease. These mixed phenotypes help explain variability in symptoms, physiologic findings, and therapeutic response [26,30].

Understanding overlap is essential for the interpretation of invasive physiology and prevents oversimplification of complex coronary pathophysiology.

### 2.4. From Biology to Physiology

**Figure 1:**
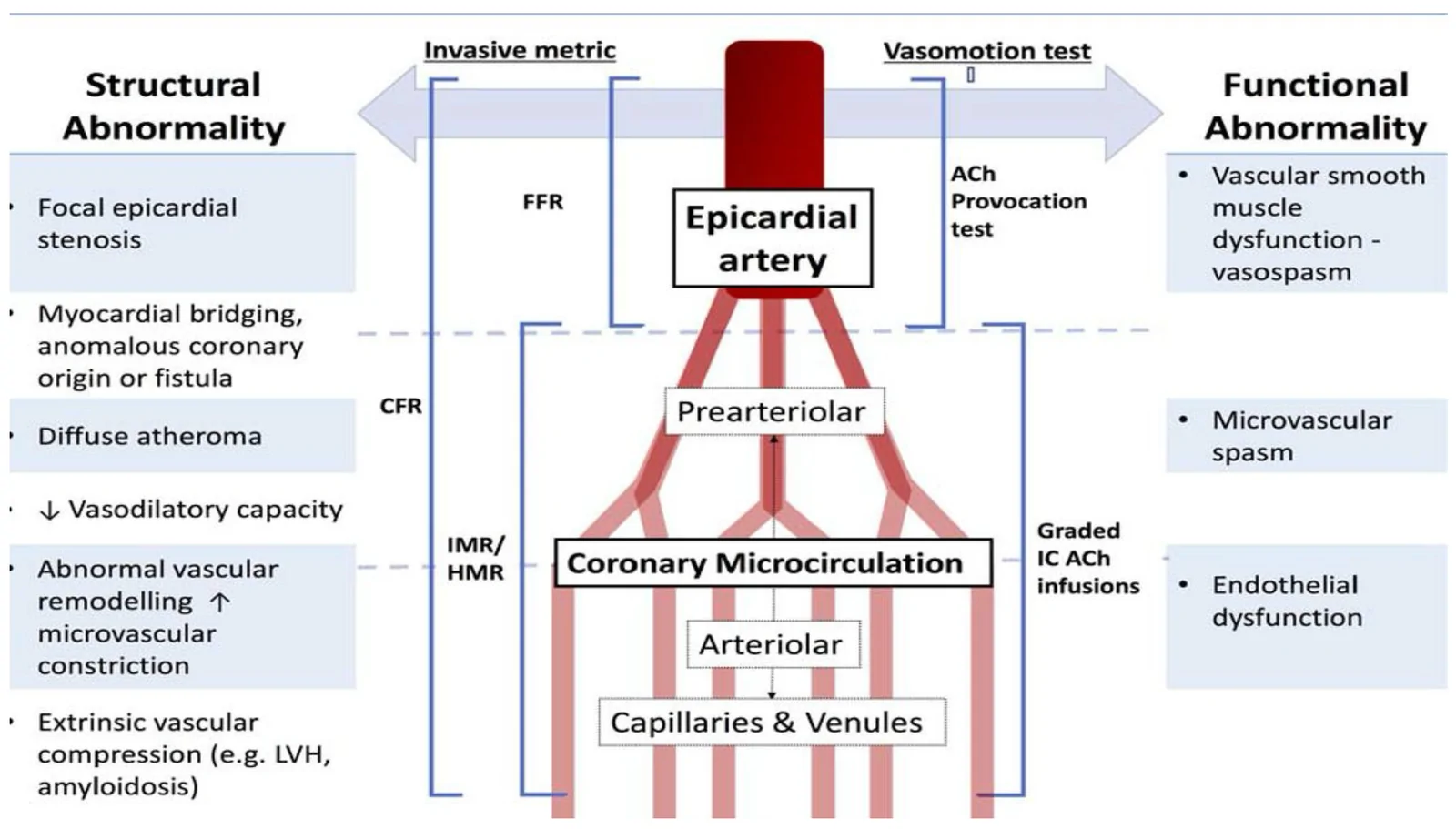
Integrated anatomic and physiologic framework illustrating structural and functional coronary vascular abnormalities across the epicardial and microvascular compartments, highlighting mechanisms not detected by angiography alone. Source: Ong P et al., 2018 [17].

Distinguishing mechanistic foundations from clinical endotypes provides the conceptual bridge to invasive coronary physiology. Functional and structural abnormalities manifest differently during physiologic testing, and appreciation of underlying biology is necessary for meaningful interpretation of coronary flow reserve, resistance indices, and vasomotor responses. The following section examines how invasive physiologic tools interrogate these endotypes and clarifies what each metric does—and does not—measure (Figure 1).

## 3. Invasive Coronary Physiology: Interrogating the Coronary Microcirculation

Invasive coronary physiology provides a means to interrogate the coronary microcirculation in vivo by quantifying distinct components of coronary hemodynamics. Unlike angiography, which describes anatomy, physiologic indices describe behavior: how blood flow responds to demand, how resistance is distributed, and how the vasculature reacts to pharmacologic or endothelial stimuli. Each metric reflects a specific aspect of coronary physiology and must be interpreted within the context of its underlying assumptions and limitations [10,11,12] (Table 1).

Critically, no single physiologic index captures the full complexity of coronary microvascular disease. The value of invasive testing lies in the integrated interpretation of complementary measures rather than reliance on any one parameter. Understanding what each index truly measures—and what it does not—is essential to avoid oversimplification and misclassification of disease mechanisms.

### 3.1. Coronary Flow Reserve: An Integrated Measure of Flow Capacity

Coronary flow reserve represents the ratio of hyperemic to resting coronary blood flow and reflects the ability of the coronary circulation to augment perfusion in response to increased metabolic demand. CFR can be measured invasively using thermodilution or Doppler techniques and noninvasively using positron emission tomography, cardiac magnetic resonance imaging, or transthoracic Doppler echocardiography [1,31,32,33].

Because CFR incorporates both resting and hyperemic flow, it reflects the combined influence of epicardial conductance, microvascular resistance, myocardial demand, and resting hemodynamics. Accordingly, it is highly sensitive to global coronary dysfunction but relatively nonspecific for the mechanism. Although thresholds vary by methodology, a CFR < 2.0 generally indicates impaired coronary flow capacity. Reduced CFR may result from elevated resting flow in conditions such as anemia, tachycardia, left ventricular hypertrophy, or heart failure and therefore does not necessarily represent primary microvascular disease. Despite this limited mechanistic specificity, reduced CFR remains a robust marker of adverse cardiovascular risk across diverse clinical populations [4,5,6,21,22,23] and should be interpreted as a global physiologic signal rather than a definitive descriptor of microvascular pathology.

However, interpretation of CFR requires caution. Elevated resting flow—driven by tachycardia, anemia, sympathetic activation, or increased myocardial demand—can reduce CFR even when maximal hyperemic flow is preserved. Conversely, preserved CFR may be observed despite underlying microvascular disease when resting flow is low or compensatory mechanisms are present. These properties explain why CFR is prognostically powerful yet mechanistically ambiguous when interpreted in isolation [10,26].

Accordingly, CFR should be viewed as a global screening index that signals impaired coronary flow reserve without reliably distinguishing between endothelial dysfunction, structural microvascular disease, or diffuse epicardial atherosclerosis.

### 3.2. Resistance-Based Indices: Defining Minimal Microvascular Resistance

Resistance-based indices were developed to address the mechanistic limitations of CFR by isolating the microcirculation under conditions of maximal vasodilation. The index of microcirculatory resistance is calculated as the product of distal coronary pressure and hyperemic mean transit time and provides an estimate of minimal achievable microvascular resistance [10,11].

By design, IMR is relatively insensitive to resting hemodynamic conditions and epicardial stenosis severity, assuming adequate hyperemia is achieved. This property allows IMR to preferentially reflect fixed resistance within the microvasculature, making it particularly useful for identifying structural CMD and severe microvascular injury [11,29].

An IMR ≥ 25 units is commonly used to define abnormal microvascular resistance in stable coronary artery disease and post-PCI settings, based on outcome and validation studies [10,11,12,29].

Context and timing are essential for IMR interpretation. In stable syndromes and ANOCA/INOCA evaluations, IMR is typically measured in the interrogated non-culprit vessel under steady-state conditions to estimate minimal microvascular resistance, and values ≥ 25 are commonly used as an abnormal threshold. In contrast, in STEMI or recent ACS, IMR is generally measured in the culprit artery immediately after reperfusion/PCI, where elevated values reflect acute microvascular injury rather than chronic microvascular remodeling. In this setting, higher thresholds (e.g., IMR ≥ 40) are best interpreted as prognostic markers of severe microvascular injury and correlate with infarct size and microvascular obstruction. Non-culprit microvascular dysfunction may also be present in ACS, reflecting more global impairment, and therefore the results should be contextualized to the clinical syndrome and hemodynamic state.

While IMR provides greater mechanistic specificity than CFR, it too has limitations. Resistance indices do not capture vasomotor instability or endothelial dysfunction and may appear normal in patients with predominantly functional CMD. Moreover, IMR reflects resistance during pharmacologic hyperemia and does not directly assess resting microvascular tone or dynamic vasomotor behavior.

### 3.3. Discordance Between CFR and Resistance Indices

Discordance between CFR and resistance-based indices is common and reflects physiologic heterogeneity rather than measurement error. Reduced CFR with normal IMR typically indicates elevated resting flow or diffuse epicardial disease rather than fixed microvascular resistance. In contrast, preserved CFR with elevated IMR suggests impaired hyperemic conductance masked by low resting flow, a phenotype associated with adverse outcomes that may be missed by CFR alone [26,30].

Recognition of discordant patterns is clinically important. Treating all abnormal CFR values as equivalent risks conflates fundamentally different mechanisms, while reliance on CFR alone may fail to identify high-risk microvascular disease. Discordance therefore provides mechanistic insight when interpreted deliberately and reinforces the need for integrated physiologic assessment rather than binary thresholds.

### 3.4. Acetylcholine Provocation Testing: Assessing Coronary Vasomotor Function

Acetylcholine provocation testing uniquely interrogates coronary vasomotor behavior by assessing endothelial-dependent responses. In healthy coronary vessels, acetylcholine stimulates endothelial nitric oxide release and induces vasodilation. In the presence of endothelial dysfunction, this response is blunted or reversed, leading to vasoconstriction [25,27,28].

Clinically, acetylcholine testing identifies both epicardial and microvascular vasospasm. Epicardial spasm is defined as a transient ≥ 90% luminal narrowing accompanied by ischemic symptoms and/or ischemic electrocardiographic changes. Microvascular spasm is defined by reproduction of ischemic symptoms and ECG changes in the absence of ≥90% epicardial constriction [25,27,28]. These distinctions are critical, as vasomotor abnormalities cannot be inferred from pressure- or resistance-based indices alone; CFR and IMR may be normal in patients with clinically significant vasospastic disease.

In practice, acetylcholine is administered using stepwise incremental intracoronary dosing according to standardized institutional protocols, with continuous ECG and hemodynamic monitoring and angiographic reassessment after each stage. Testing should be avoided in the presence of severe obstructive disease in the interrogated vessel, significant left main stenosis, unstable hemodynamics, or when sustained spasm cannot be managed safely. Prompt relief with intracoronary nitrates supports the diagnosis and confirms reversibility.

Contemporary data indicate that acetylcholine testing is safe when performed in experienced centers; however, variability in dosing strategies, infusion versus bolus techniques, and interpretation thresholds across institutions remains an important limitation. Accordingly, results should be interpreted within institutional standards and integrated with complementary physiologic indices rather than considered in isolation [14,15,16,25,27,28].

### 3.5. Integrated Physiologic Interpretation

No single physiologic metric captures the full spectrum of coronary microvascular dysfunction. CFR identifies impaired flow reserve, resistance-based indices define minimal microvascular conductance, and acetylcholine testing assesses vasomotor stability. When interpreted together and in a clinical context, these measures allow physiologic characterization of CMD endotypes without redundancy.

**Figure 2:**
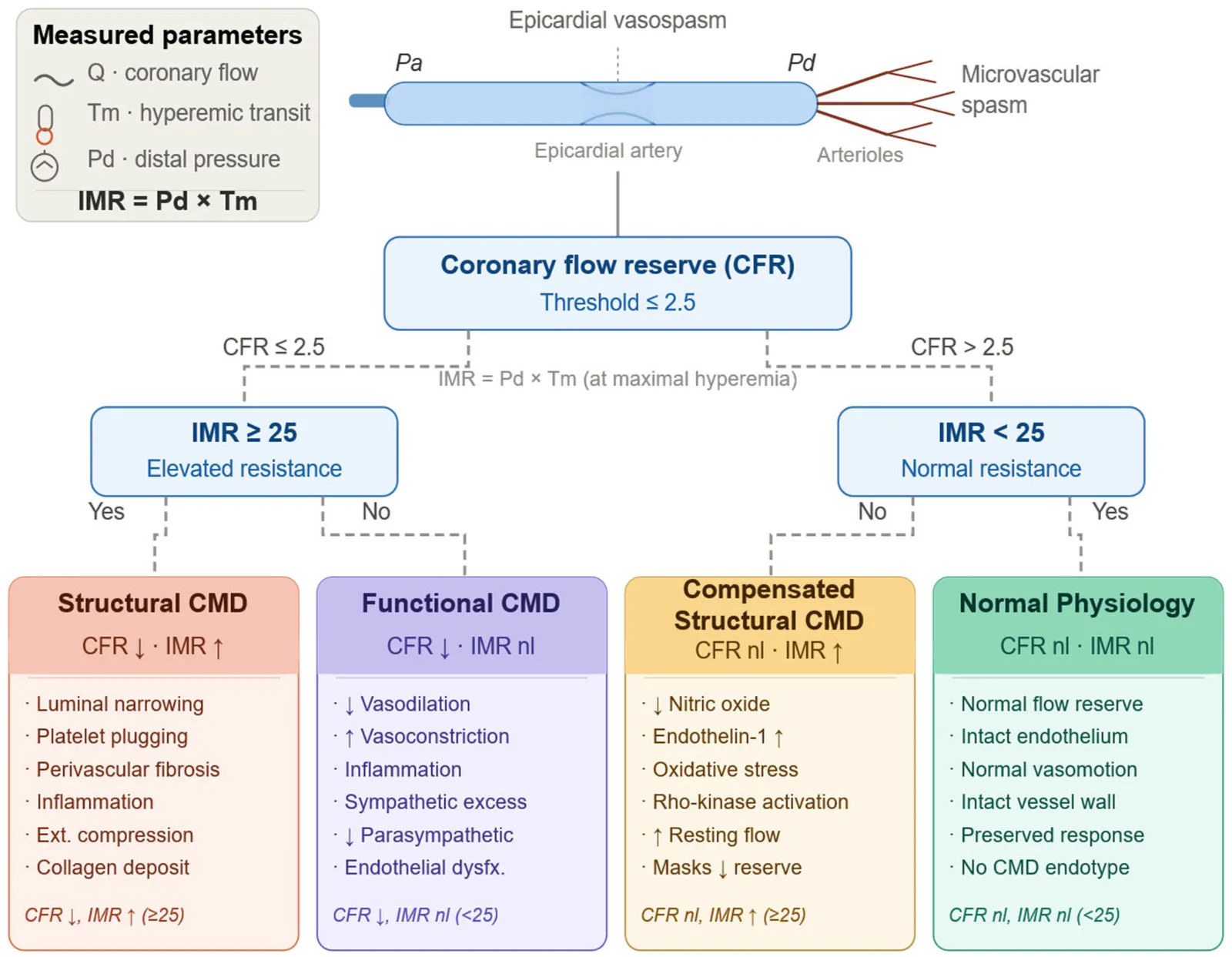
Invasive physiologic framework for classification of coronary microvascular dysfunction (CMD) by coronary flow reserve (CFR) and index of microcirculatory resistance (IMR). Following exclusion of obstructive epicardial disease, CFR identifies impaired vasodilatory capacity, and IMR differentiates structural from functional CMD endotypes. Elevated IMR with preserved CFR identifies compensated structural CMD. Normal CFR and IMR are consistent with normal microvascular physiology. CFR = coronary flow reserve; CMD = coronary microvascular dysfunction; IMR = index of microcirculatory resistance; Pd = distal coronary pressure; Tm = hyperemic mean transit time; nl = normal limits; ↑ = elevated; ↓ = reduced.

This integrative approach reflects the broader philosophy of contemporary coronary physiology: invasive testing is most valuable when it clarifies mechanism rather than merely confirms ischemia or assigns binary diagnoses [10,26,30] (Figure 2).

## 4. Practical Cath-Lab Workflow for Invasive Assessment of Coronary Microvascular Dysfunction

A structured approach is essential for the effective application of invasive microvascular testing in the cardiac catheterization laboratory. Without a clear workflow, physiologic assessment risks becoming fragmented or redundant, limiting its diagnostic and clinical value. Contemporary guideline documents and expert consensus statements emphasize that microvascular testing should be performed selectively, guided by clinical context, and interpreted within an integrated physiologic framework rather than as a series of isolated measurements [14,15,16].

### 4.1. Patient Selection and Clinical Context

Appropriate patient selection is the first critical step. Invasive microvascular testing is most informative in patients with persistent angina or objective ischemia that is not adequately explained by angiographic findings. Common scenarios include patients with angina and nonobstructive coronary arteries, individuals with diffuse or intermediate epicardial disease without focal flow-limiting stenoses, and patients with persistent or recurrent symptoms following apparently successful PCI [14,15,16].

**Figure 3:**
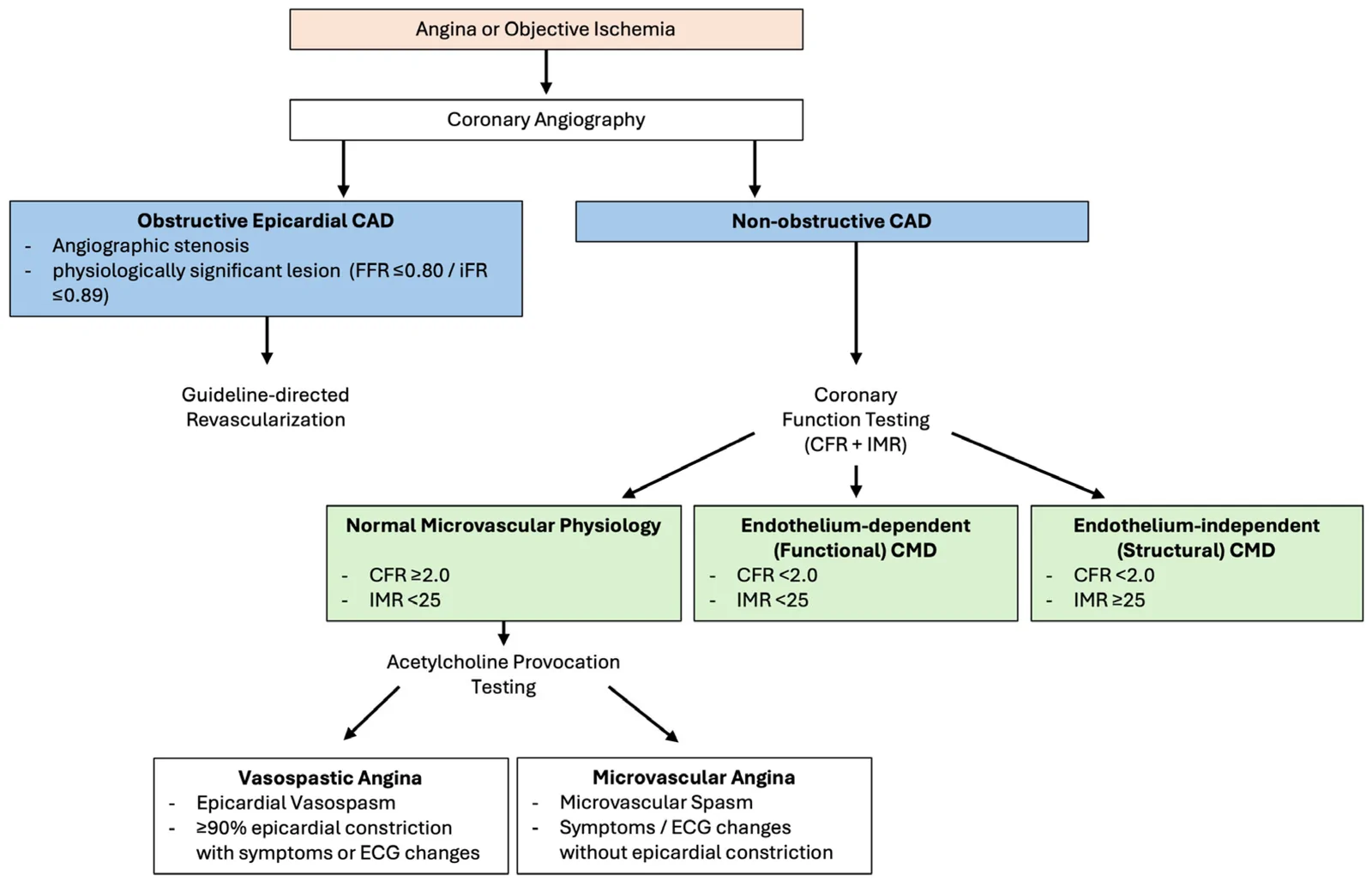
Physiology-guided diagnostic framework for the evaluation of patients with suspected coronary microvascular dysfunction. Patients presenting with angina or objective evidence of ischemia are first assessed for obstructive epicardial coronary artery disease using angiographic findings and physiologic indices, including fractional flow reserve (FFR ≤ 0.80) or instantaneous wave-free ratio (iFR ≤ 0.89). In the absence of flow-limiting epicardial disease, coronary function testing integrating coronary flow reserve (CFR) and the index of microcirculatory resistance (IMR) enables classification of microvascular physiology. Distinct patterns of CFR and IMR differentiate normal microvascular physiology, endothelium-dependent (functional) dysfunction, and endothelium-independent (structural) coronary microvascular disease, providing a framework for mechanism-guided diagnosis and clinical decision-making in patients with non-obstructive coronary artery disease.

In contrast, routine microvascular testing is generally not indicated in patients with clearly flow-limiting epicardial stenoses requiring revascularization or in those with advanced noncardiac comorbidities where results are unlikely to influence management. Establishing the clinical question in advance—diagnostic clarification, symptom explanation, or prognostic assessment—helps determine the appropriate testing strategy (Figure 3).

### 4.2. Step 1: Exclusion of Flow-Limiting Epicardial Disease

Assessment should begin with exclusion of functionally significant epicardial coronary artery disease using fractional flow reserve or non-hyperemic pressure ratios. This step ensures that ischemia attributable to focal epicardial stenosis is identified and appropriately treated before attributing symptoms to microvascular disease [14,15,16].

Only after epicardial flow-limiting disease has been excluded or treated should microvascular assessment be pursued. This sequencing prevents misclassification and avoids conflating epicardial and microvascular mechanisms.

### 4.3. Step 2: Assessment of Coronary Flow Reserve and Microvascular Resistance

Following exclusion of epicardial disease, evaluation of microvascular function typically begins with measurement of coronary flow reserve and a resistance-based index. CFR provides an integrated assessment of coronary flow capacity, while resistance indices such as IMR provide mechanistic specificity by estimating minimal achievable microvascular resistance [10,11,12].

Operationally, CFR values < 2.0–2.5 and IMR values ≥ 25 units are commonly used thresholds to identify abnormal microvascular function in stable coronary artery disease, recognizing that values exist on a continuum and must be interpreted in a clinical context [10,11,12,29]. Discordance between CFR and IMR should prompt consideration of underlying physiologic heterogeneity rather than being dismissed as technical variability [26,30].

Importantly, measurement of CFR and IMR can usually be performed efficiently during the same procedural sequence, minimizing additional procedural time and contrast exposure when performed by experienced operators.

### 4.4. Step 3: Vasomotor Assessment with Acetylcholine

When vasomotor dysfunction is suspected—particularly in patients with episodic angina, rest symptoms, or ischemia disproportionate to exertional demand—acetylcholine provocation testing provides complementary information that cannot be inferred from flow- or resistance-based indices [25,27,28].

Acetylcholine testing should be performed using standardized protocols with careful monitoring for symptoms, electrocardiographic changes, and angiographic responses. Identification of epicardial or microvascular spasm has direct therapeutic implications and may explain symptoms in patients with otherwise normal pressure- and flow-derived indices [14,15,16].

While concerns regarding safety have historically limited adoption, contemporary studies demonstrate that acetylcholine testing is safe when performed in experienced centers using established protocols [25,27,28].

### 4.5. Integration and Reporting

The diagnostic yield of invasive microvascular testing depends on integrated interpretation rather than individual thresholds. Findings should be reported in a structured manner that links physiologic results to dominant CMD endotypes and clinical presentation. For example, reduced CFR with elevated IMR suggests structural CMD, whereas abnormal acetylcholine responses with preserved resistance indices indicate vasomotor dysfunction.

Clear documentation of physiologic findings facilitates communication with referring clinicians and supports mechanism-directed management. This approach aligns with SCAI quality initiatives emphasizing standardized reporting and clinically actionable interpretation of coronary physiology [16].

### 4.6. Workflow Considerations and Feasibility

When incorporated thoughtfully, invasive microvascular testing can be performed with acceptable procedural duration and low complication rates. Key determinants of feasibility include operator familiarity, team coordination, and institutional protocols. Selective rather than routine application helps maximize yield while minimizing unnecessary testing [14,15,16].

## 5. Clinical Implications of Invasive Microvascular Testing in Stable Coronary Artery Disease

In stable coronary artery disease, invasive microvascular testing has its greatest clinical impact in patients whose symptoms or ischemic burden are not adequately explained by angiographic findings alone. This includes individuals with angina and nonobstructive coronary arteries, patients with diffuse or moderate epicardial disease without focal flow-limiting stenoses, and those with persistent symptoms following apparently successful revascularization. Across these populations, CMD is prevalent, prognostically relevant, and frequently under-recognized [4,5,6,14].

Rather than serving as a confirmatory test, invasive microvascular assessment reframes ischemia as a disorder of coronary physiology rather than anatomy. By identifying dominant mechanisms of dysfunction, testing provides diagnostic clarity and supports a more individualized approach to management.

### 5.1. Angina with Nonobstructive Coronary Arteries (ANOCA/INOCA)

Patients with angina and nonobstructive coronary arteries represent one of the most common and challenging populations encountered in contemporary practice. Historically, the absence of obstructive epicardial disease on angiography often led to reassurance or empiric treatment without a clear mechanistic diagnosis. However, accumulating evidence demonstrates that this approach underestimates both symptom burden and cardiovascular risk [4,5,6].

The WISE program fundamentally altered the understanding of this population by demonstrating that impaired coronary flow reserve and endothelial dysfunction are associated with adverse cardiovascular outcomes, including myocardial infarction and heart failure hospitalization, despite the absence of obstructive coronary artery disease [4,5,6]. These findings established CMD as a clinically meaningful substrate of ischemia rather than a benign or incidental finding.

Invasive coronary function testing has since demonstrated that a substantial proportion of ANOCA/INOCA patients exhibit definable physiologic abnormalities, including endothelial-dependent CMD, structural microvascular disease, and vasospastic disorders [14,15,16,25,27,28]. Importantly, these phenotypes are heterogeneous and not interchangeable. Patients with endothelial dysfunction may experience episodic symptoms at rest, whereas those with structural CMD more often report exertional angina with reproducible ischemia.

The ILIAS-ANOCA trial further demonstrated that ad hoc invasive coronary function testing at the time of angiography is feasible and safe, and that incorporation of mechanism-directed therapy improves angina-related health status compared with usual care [34].

Parallel advances in noninvasive phenotyping further support this framework. Quantitative stress perfusion CMR has been shown to improve diagnostic attribution and angina-related quality of life when incorporated into clinical decision-making, highlighting the complementary role of noninvasive flow assessment within a physiology-based approach [35].

These data align with a patient-centered framework in which angina is recognized as the potential consequence of coexisting epicardial atherosclerosis and functional coronary disorders, and management is tailored to the dominant physiologic endotype rather than anatomy alone.

### 5.2. Sex-Specific Considerations in Stable CAD

Sex differences are among the most consistent findings in CMD research. Women presenting with angina are more likely than men to have nonobstructive coronary arteries and a higher prevalence of microvascular and vasomotor dysfunction [5,36]. These differences likely reflect a combination of biological factors, including differences in microvascular structure, hormonal influences, inflammatory signaling, and patterns of diffuse atherosclerosis, as well as systemic and health-care–related factors.

Clinically, these differences contribute to delayed diagnosis, under-recognition of ischemic heart disease, and persistent symptom burden in women. Invasive functional testing offers an opportunity to mitigate these disparities by providing objective evidence of coronary dysfunction and guiding mechanism-directed therapy [14,15,16].

Recognition of CMD in women with angina challenges traditional paradigms and underscores the importance of moving beyond angiographic definitions of coronary artery disease. Importantly, CMD-related ischemia in women is associated with adverse outcomes and should not be dismissed as low risk [4,5,6].

Although CMD is more prevalent in women with ANOCA/INOCA, sex-specific thresholds for physiologic indices and provocation testing remain insufficiently validated, and current invasive strategies are generally applied similarly across sexes.

### 5.3. CMD in Diffuse and Moderate Epicardial Disease

CMD frequently coexists with diffuse or moderate epicardial atherosclerosis that does not produce focal flow-limiting stenoses. In such cases, angiography may underestimate ischemic burden, while focal physiologic assessment alone may fail to capture global coronary dysfunction.

Invasive assessment of coronary flow reserve and microvascular resistance can identify impaired flow capacity in this setting and explain ischemia that appears disproportionate to angiographic severity [10,11,12]. Recognition of concomitant CMD is particularly relevant when considering revascularization strategies, as focal intervention may not address the dominant physiologic mechanism.

### 5.4. Persistent or Recurrent Angina After PCI

Persistent angina following apparently successful PCI is common and often perplexing. Angiography may demonstrate patent stents and no residual focal disease, leading to uncertainty regarding symptom etiology and, in some cases, repeated invasive procedures.

Invasive physiologic studies demonstrate that residual angina after PCI is frequently attributable to underlying CMD rather than procedural failure or restenosis [29,37]. Structural microvascular disease, endothelial dysfunction, and diffuse atherosclerosis may all contribute to impaired flow reserve despite normalization of epicardial pressure gradients.

In this context, invasive microvascular testing reframes post-PCI angina as a manifestation of persistent coronary pathophysiology rather than inadequate revascularization. This distinction is clinically important, as additional stenting is unlikely to provide benefit when symptoms are driven by microvascular mechanisms.

### 5.5. Prognostic and Therapeutic Implications

Beyond symptom attribution, CMD carries important prognostic implications. Reduced coronary flow reserve and elevated microvascular resistance have been associated with increased risk of major adverse cardiovascular events independent of epicardial disease severity [4,5,6,29]. These findings highlight the importance of recognizing CMD as a marker of global coronary risk.

The therapeutic implications of invasive microvascular testing are evolving. The CorMicA trial demonstrated that invasive coronary function testing followed by stratified, mechanism-directed therapy improved angina severity and quality of life compared with usual care in patients with angina and nonobstructive coronary arteries [33]. While broader outcomes data remain limited, CorMicA provides proof-of-concept that physiologic phenotyping can translate into meaningful clinical benefit. However, its modest sample size, single-center design, and lack of hard clinical endpoints limit definitive conclusions regarding long-term cardiovascular outcomes.

Importantly, invasive testing does not mandate specific therapies but rather informs rational selection of existing treatments and avoids unnecessary or ineffective interventions. As experience grows and evidence evolves, mechanism-based management strategies may become an increasingly central component of care for patients with stable coronary artery disease.

## 6. Coronary Microvascular Dysfunction and Microvascular Obstruction After Myocardial Infarction

Although the term “microvascular dysfunction” is used across both stable coronary syndromes and acute myocardial infarction, these entities differ fundamentally in biology and clinical implications. In stable syndromes, CMD reflects chronic abnormalities of vasomotor regulation or structural remodeling of the microcirculation. In contrast, following myocardial infarction, microvascular impairment primarily represents acute microvascular injury or obstruction, with predominantly prognostic rather than diagnostic significance.

Coronary microvascular dysfunction following acute myocardial infarction represents a biologically distinct entity that differs fundamentally from chronic microvascular disease observed in stable coronary syndromes. In this setting, microvascular impairment arises predominantly from acute injury to the coronary microcirculation, rather than long-standing endothelial dysfunction or fixed structural remodeling. Importantly, restoration of epicardial coronary patency does not ensure effective myocardial reperfusion, and persistent microvascular injury remains a major determinant of infarct size, adverse ventricular remodeling, and long-term clinical outcomes [38,39,40].

### 6.1. Pathophysiology of Acute Microvascular Injury

Acute microvascular dysfunction after myocardial infarction is driven by a convergence of mechanisms that collectively impair tissue-level perfusion. Distal embolization of thrombotic and atherosclerotic debris during plaque rupture and percutaneous coronary intervention contributes to microvascular plugging, while ischemia–reperfusion injury induces endothelial swelling, capillary destruction, and increased vascular permeability. These processes are further amplified by inflammatory cell infiltration, oxidative stress, and extravascular compression from myocardial edema within the infarct zone [38,39]. The result is a heterogeneous and spatially variable disruption of microvascular flow that is poorly reflected by angiographic indices of epicardial reperfusion.

This dissociation explains why angiographic success, including achievement of Thrombolysis in Myocardial Infarction (TIMI) grade 3 flow, frequently fails to translate into effective myocardial salvage. In this context, microvascular injury represents the final common pathway linking epicardial occlusion, reperfusion therapy, and downstream myocardial damage.

### 6.2. Microvascular Obstruction

Microvascular obstruction (MVO) represents the most severe manifestation of post-infarction microvascular injury and reflects irreversible damage to the coronary microcirculation within the infarct core. MVO is best characterized using cardiac magnetic resonance imaging (CMR) with late gadolinium enhancement, where it appears as a hypo-enhanced region within the infarcted myocardium [38,41]. The presence and extent of MVO are strongly associated with larger infarct size, impaired myocardial recovery, adverse left ventricular remodeling, heart failure hospitalization, and increased mortality, independent of epicardial revascularization success [38,40,41].

From a mechanistic standpoint, MVO reflects a combination of capillary destruction, intraluminal obstruction, endothelial disruption, and extravascular compression that collectively prevent effective reperfusion at the tissue level. Clinically, MVO highlights the limitations of angiography-based assessment and underscores the importance of microvascular integrity as a determinant of myocardial recovery. Despite contemporary reperfusion strategies, MVO remains prevalent and continues to account for substantial residual risk following acute myocardial infarction.

### 6.3. Invasive Assessment of Microvascular Resistance After Myocardial Infarction

Invasive coronary physiology provides a complementary approach to characterizing microvascular injury in the acute and early post-infarction period. Among available indices, the IMR has emerged as a robust, reproducible, and relatively load-independent marker of microvascular injury following primary percutaneous coronary intervention. Elevated IMR measured immediately after reperfusion correlates closely with the presence and extent of microvascular obstruction on CMR and independently predicts adverse left ventricular remodeling, heart failure, and mortality [11,29,41].

In the acute myocardial infarction setting, markedly elevated IMR values—particularly thresholds of ≥40—have been associated with severe microvascular injury, extensive microvascular obstruction, larger infarct size, and increased risk of subsequent heart failure and death [34]. This higher threshold reflects the severity of post-infarction microcirculatory damage and should be interpreted as a prognostic marker of irreversible microvascular injury, rather than as evidence of vasomotor dysfunction or a target for intracoronary vasodilator therapy.

In contrast to stable coronary syndromes, coronary flow reserve is less reliable in the acute myocardial infarction setting, owing to dynamic alterations in resting coronary flow, myocardial stunning, and neurohumoral activation [13,39]. Accordingly, invasive microvascular assessment after myocardial infarction should be viewed primarily as a risk-stratification and prognostic tool, distinguishing it conceptually from its diagnostic role in stable coronary disease.

Interpretation of IMR thresholds differs significantly between stable coronary disease and acute myocardial infarction. While an IMR ≥ 25 is commonly used to define abnormal microvascular resistance in stable settings, higher thresholds in the context of myocardial infarction reflect a distinct pathophysiologic process. Specifically, IMR ≥ 40 measured in the culprit vessel immediately following primary percutaneous coronary intervention is associated with severe microvascular injury and carries prognostic significance rather than diagnostic utility. In this setting, IMR reflects the extent of irreversible microvascular damage rather than functional vasomotor abnormalities. Importantly, microvascular dysfunction may also be present in non-culprit vessels due to systemic inflammatory and neurohumoral responses, highlighting the need for careful contextual interpretation of physiologic indices in acute coronary syndromes.

### 6.4. Risk Stratification

Beyond prognostication, invasive assessment of microvascular injury provides a physiologic framework for post–myocardial infarction risk stratification. Patients with extensive MVO or markedly elevated IMR represent a high-risk subgroup prone to adverse ventricular remodeling and heart failure progression, despite successful epicardial revascularization [29,40,41]. Identification of severe microvascular injury may therefore support closer clinical surveillance, earlier optimization of guideline-directed medical therapy, and consideration for enrollment in clinical trials targeting reperfusion injury and myocardial remodeling.

Importantly, elevated IMR reflects fixed microvascular injury rather than vasomotor dysfunction and should not be interpreted as an indication for intracoronary vasodilator therapy. In contrast to functional microvascular disorders, post-infarction microvascular obstruction is unlikely to respond to agents such as calcium channel blockers or adenosine, underscoring the distinct pathobiology of post-myocardial infarction microvascular disease [35,38,42]. At present, no microvascular-targeted therapy has consistently demonstrated improved clinical outcomes following myocardial infarction; however, IMR offers a biologically grounded tool for refining prognosis, identifying vulnerable patients, and supporting the development of mechanistically informed therapeutic strategies. These observations underscore the need to integrate coronary microvascular assessment into a broader clinical framework that spans stable coronary syndromes, acute ischemic presentations, and longitudinal risk stratification.

## 7. Limitations, Implementation Challenges, and Unresolved Questions

Despite increasing recognition and expanding guideline support for invasive coronary function testing, important limitations and practical barriers remain. Current invasive tools provide indirect functional measurements rather than direct structural assessment of the microcirculation. As a result, physiologic indices require contextual interpretation and should be integrated across modalities rather than applied as isolated diagnostic labels [10,26,30].

Diagnostic thresholds for CFR and resistance-based indices vary by methodology and clinical setting, and values exist on a spectrum rather than as binary cutoffs [10,11,12,31,32,33]. Variability in hyperemic protocols and resting hemodynamic conditions further complicates interpretation, underscoring the need for harmonized testing strategies and reproducible protocols [14,15,16].

Practical implementation also remains uneven. Operator experience, inter-center differences in acetylcholine dosing and interpretation, procedural time, and reimbursement considerations continue to limit broader adoption [14,15,16,25,27,28]. Although safety is well established in experienced centers, wider dissemination will require structured training pathways and standardized workflow integration [25,27,28].

Therapeutic evidence continues to evolve. CorMicA and the ILIAS ANOCA trial demonstrate that mechanism-directed strategies improve angina-related health status [33,34], yet data demonstrating a reduction in major adverse cardiovascular events are limited. Similarly, stratified treatment approaches in related syndromes such as MINOCA (e.g., PROMISE) support the principle of tailored care but highlight the need for adequately powered outcome-driven trials [42].

Finally, key questions remain regarding the role of invasive microvascular testing in cardiometabolic disease, HFpEF, and acute coronary syndromes [21,22,23,41]. In ACS, invasive assessment is primarily prognostic, and targeted microvascular therapies have not consistently improved outcomes [29,40,41]. Future investigation should focus on standardized physiologic algorithms and randomized trials stratified by CMD endotype to determine whether physiology-guided care translates into durable clinical benefit.

## 8. Conclusions

CMD represents a fundamental, yet historically underappreciated, contributor to myocardial ischemia across the spectrum of coronary artery disease. Limitations of angiography-centered diagnostic strategies have increasingly become apparent, particularly in patients with angina and nonobstructive coronary arteries, persistent symptoms after revascularization, and acute coronary syndromes complicated by microvascular injury.

Invasive coronary physiology provides a unique window into coronary microvascular behavior, enabling assessment of flow reserve, microvascular resistance, and vasomotor function that cannot be inferred from anatomy alone. When interpreted within a structured, mechanism-based framework, these tools allow clinicians to characterize CMD endotypes, clarify the pathophysiology underlying ischemic symptoms, and distinguish prognostic insight from therapeutic opportunity.

Importantly, invasive microvascular testing should not be viewed as an isolated diagnostic exercise or a substitute for clinical judgment. Its value lies in integration—linking coronary biology, physiologic behavior, and patient presentation to inform rational, individualized care. As guideline support grows and clinical experience expands, invasive microvascular assessment is poised to complement epicardial physiology within a comprehensive approach to coronary disease that prioritizes mechanism over morphology.

Future progress will depend on continued refinement of physiologic tools, development of targeted therapies, and generation of outcomes-based evidence that defines how best to translate mechanistic insight into durable clinical benefit. Until then, invasive microvascular testing offers a powerful means of understanding coronary disease beyond the angiogram and represents an important step toward more precise, physiology-driven cardiovascular care.

## Figures and Tables

**Table 1:** Physiologic patterns associated with major mechanisms of myocardial ischemia. Integrated interpretation of coronary flow reserve (CFR), index of microcirculatory resistance (IMR), and acetylcholine provocation testing allow differentiation between obstructive epicardial coronary artery disease, structural coronary microvascular dysfunction, vasospastic disorders, and acute microvascular injury following myocardial infarction.

Physiologic Pattern	CFR	IMR	Acetylcholine Response	Mechanistic Interpretation	Clinical Context
Epicardial flow-limiting stenosis	↓ (<2.0)	Normal (<25)	Normal	Obstructive epicardial coronary artery disease	Stable CAD, focal stenosis
Structural CMD	↓ (<2.0)	↑ (>25)	Normal	Fixed microvascular disease with increased minimal resistance	Cardiometabolic disease, diffuse CAD
Endothelial CMD	Normal or ↓	Normal	Abnormal vasoconstrictive response	Endothelial dysfunction with impaired vasodilatory signaling	ANOCA/INOCA
Vasospastic angina	Normal	Normal	≥90% epicardial constriction with symptoms/ECG changes	Dynamic epicardial vasospasm	Rest angina
Post-MI microvascular injury	Variable	↑ (>40)	Not typically tested	Severe microvascular injury and microvascular obstruction	STEMI after PCI

Abbreviations: CFR, coronary flow reserve; IMR, index of microcirculatory resistance; CMD, coronary microvascular dysfunction. ↓, decreased/reduced; ↑, increased/elevated.

## Data Availability

No new data were created or analyzed in this study. Data sharing is not applicable to this article.
